# Computational Methods in Cooperation with Experimental Approaches to Design Protein Tyrosine Phosphatase 1B Inhibitors in Type 2 Diabetes Drug Design: A Review of the Achievements of This Century

**DOI:** 10.3390/ph15070866

**Published:** 2022-07-14

**Authors:** Mara Ibeth Campos-Almazán, Alicia Hernández-Campos, Rafael Castillo, Erick Sierra-Campos, Mónica Valdez-Solana, Claudia Avitia-Domínguez, Alfredo Téllez-Valencia

**Affiliations:** 1 Facultad de Medicina y Nutrición, Universidad Juárez del Estado de Durango, Avenida Universidad y Fanny Anitúa S/N, Durango 34000, Mexico; maraicamp@gmail.com; 2 Facultad de Química, Departamento de Farmacia, Universidad Nacional Autónoma de México, Ciudad de Mexico 04510, Mexico; hercam@unam.mx (A.H.-C.); rafaelc@unam.mx (R.C.); 3 Facultad de Ciencias Químicas, Universidad Juárez del Estado de Durango Campus Gómez Palacio, Avenida Artículo 123 S/N, Fracc, Filadelfia, Gómez Palacio 35010, Mexico; ericksier@gmail.com (E.S.-C.); valdezandyval@gmail.com (M.V.-S.)

**Keywords:** PTP1B inhibitors, computer-assisted drug design, molecular docking, virtual screening, pharmacophore modeling, QSAR, type 2 diabetes

## Abstract

Protein tyrosine phosphatase 1B (PTP1B) dephosphorylates phosphotyrosine residues and is an important regulator of several signaling pathways, such as insulin, leptin, and the ErbB signaling network, among others. Therefore, this enzyme is considered an attractive target to design new drugs against type 2 diabetes, obesity, and cancer. To date, a wide variety of PTP1B inhibitors that have been developed by experimental and computational approaches. In this review, we summarize the achievements with respect to PTP1B inhibitors discovered by applying computer-assisted drug design methodologies (virtual screening, molecular docking, pharmacophore modeling, and quantitative structure–activity relationships (QSAR)) as the principal strategy, in cooperation with experimental approaches, covering articles published from the beginning of the century until the time this review was submitted, with a focus on studies conducted with the aim of discovering new drugs against type 2 diabetes. This review encourages the use of computational techniques and includes helpful information that increases the knowledge generated to date about PTP1B inhibition, with a positive impact on the route toward obtaining a new drug against type 2 diabetes with PTP1B as a molecular target.

## 1. Introduction

Cancer, obesity, and diabetes mellitus are major health problems worldwide, causing around 9.6, 2.8, and 1.6 million deaths a year, respectively [1]. In addition, all of these disease impose a high expense on the health system and even more when they coexist [2]. Despite the wide variety of oral and injectable therapies available for these diseases, their uses are limited by efficacy, adverse effects, and contraindications [3,4,5,6,7]. Therefore, the increased prevalence of these diseases highlights the necessity of searching for new drugs for their treatment.

**Figure 1:**
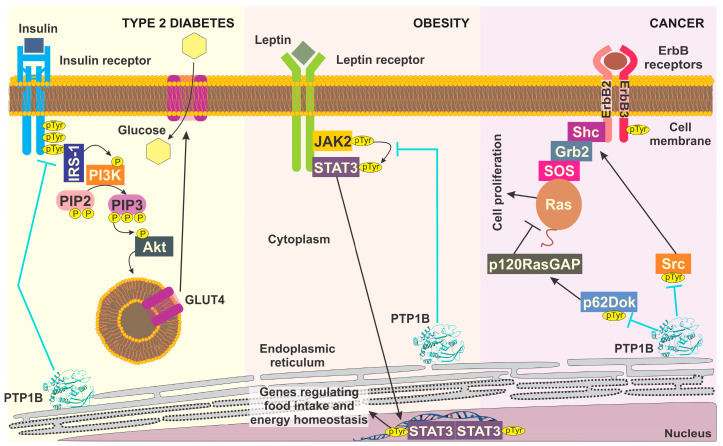
Role of PTP1B in the signal pathway for type 2 diabetes, obesity, and cancer.

In this sense, protein tyrosine phosphatase 1B (PTP1B) has been established as a pharmacological target for these pathologies. Its substrates are involved in multiple cellular processes, such as glucose homeostasis regulated by insulin signaling, decreased food intake, increased energy expenditure, cellular proliferation, and more. PTP1B is considered an interesting drug target for the treatment of type 2 diabetes because it dephosphorylates the insulin receptor (IR) and the insulin receptor substrate 1 (IRS-1), inactivating the downstream pathway of phosphatidylinositol 3-kinase (PI3K)-Akt and preventing the translocation of glucose transporter 4 (GLUT4) [8,9] (Figure 1). In addition, attention has been on PTP1B as a potentially excellent therapeutic target in obesity, owing to the role that it plays in the regulation of leptin signaling. PTP1B downregulates leptin receptor (LR) stimuli by disrupting the autophosphorylation of JAK2 and subsequent LR phosphorylation, as well as the activation of STAT3, which mediates the transcription of target genes [10,11] (Figure 1). On the other hand, it has been reported that PTP1B can block the induction of cell proliferation and cell survival via the negative regulation of the signaling cascade linking ErbB2-PTP1B-Src kinase, which contributes to the tumor-suppressor function of PTP1B in cancer cells [12,13,14] (Figure 1). However, this last role of PTP1B has become increasingly controversial; for an extensive review, the reader is referred to [15,16]. The above-mentioned controversy highlights the relevance of developing therapies targeting PTP1B in these diseases.

Accordingly, the development of PTP1B inhibitors has increased considerably for the treatment of these diseases. Nevertheless, PTP1B inhibitors have not progressed beyond the preclinical stage due to bioavailability and specificity challenges [17,18]. One of the principal hindrances is the chemical environment of the PTP1B active site, which is highly polar; therefore, it attracts negatively charged molecules with poor membrane permeability and limited oral bioavailability [19]. Regarding selectivity, the PTP family is characterized by an exceptionally high degree of sequence conservation across active sites [20]. T-cell protein tyrosine phosphatase (TCPTP) is the closest homolog of PTP1B [21,22]. It has been reported that mice lacking TCPTP die 5 weeks after birth due to defects in immune function and hematopoiesis failure [23]. In contrast, PTP1B knockout mice were reported to have a normal life span [24]. Therefore, it is important to consider these challenges in the design of PTP1B inhibitors.

Moreover, drug development is a costly and complex process that consumes a lot of time—around 10–15 years [25,26]. Recently, computer-assisted drug design (CADD) has become a crucial component used by the pharmaceutical industry and academic institutions to decrease costs and is a huge time saver due to the minimization of laboratory work and experimentation. With this review, we aim to present the efforts made to obtain PTP1B inhibitors through CADD, with particular focus on the protein sites used to this end, as well as the computational strategy, supported by the inhibitory activity evaluated in vitro. Our review included only articles published during this century and focused on PTP1B as a target for type 2 diabetes drug design. The research was carried out using various groups of keywords, such as PTP1B inhibitors, virtual screening, molecular docking, pharmacophore modeling, 3D-QSAR, and computational drug design.

## 2. PTP1B Structure and Target Sites

**Figure 2:**
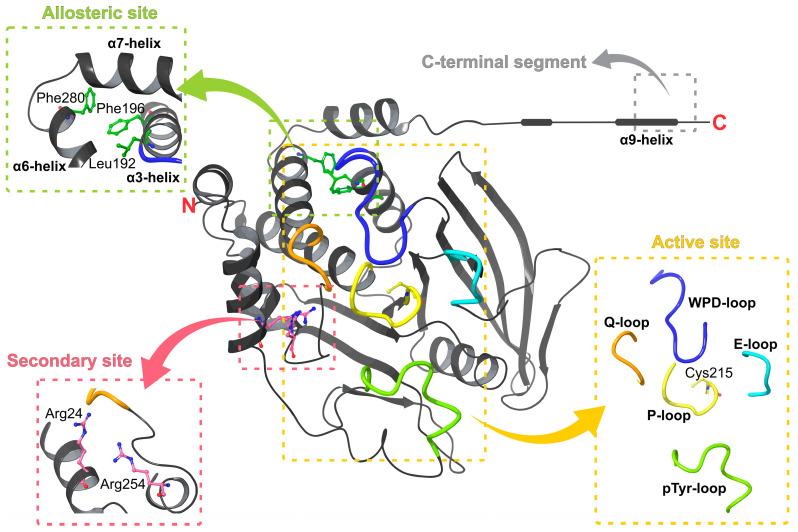
PTP1B catalytic domain structure. The image highlights the different sites where inhibitors have been reported.

PTP1B, a ubiquitously expressed classical non-receptor PTP, is encoded by the PTPN1 gene. This enzyme hydrolyzes phosphotyrosine (pTyr)-containing proteins. In the cell, it is a protein of ~50 kDa (435 amino acids) located in the endoplasmic reticulum; however, it was originally isolated from a human placenta as a 37 kDa protein that includes the catalytic domain (residues 1–321) [27,28,29]. Structurally, the enzyme is formed by an N-terminal catalytic domain, two proline-rich sequences, and a C-terminal hydrophobic region (35 residues), which serves to attach the enzyme to the membrane of the endoplasmic reticulum [28,30,31]. Furthermore, it contains a regulatory segment of ~115 residues on the C-terminal side of the catalytic domain [27] (Figure 2).

PTP1B was the first PTP structure reported at high resolution [32]. To date, 274 human PTP1B structures have been deposited in the Protein Data Bank (PDB, www.rcsb.org, accessed on 1 February 2022). Of these, around two hundred twenty-five structures are in complex with different ligands: nine in the apo form and the rest with some mutations or chemical modifications, all with a resolution ranging from 1.5 to 3.3 Å. Most crystallographic complexes are formed with competitive inhibitors bound at the PTP1B active site. Nevertheless, there are some complexes with inhibitors bound at distinct sites, such as the C-terminal region of the catalytic domain (helix α9) [33] and at a site formed by helices α3, α6, and α7 called an allosteric site [34] (Figure 2).

### 2.1. Active and Secondary Sites

The PTP1B active site is formed by several loop regions (P-, WPD-, Q-, E-, and pTyr loops) and a secondary site (Figure 2). The phosphate-binding loop, i.e., the P loop or PTP loop (residues 214–221), contains the conserved signature motif VHCSXGXGR[T/S]G, including the catalytic Cys215 and the invariant Arg221, which provides specificity for pTyr in classical PTPs [20,35]. The WPD loop (residues 179 to 189) acts as a flexible gate to the active site that can take closed (active) and open (inactive) conformations, where substrate binding only occurs when the loop is in the open state [35,36]. Another loop is the Q loop (residues 262 to 266), which contains conserved Glu262 that coordinates the water molecule necessary for hydrolysis of the thiophosphate intermediate [35]. The E loop contains multiple conserved residues; it has been suggested that this loop coordinates the dynamics of the WPD loop [37]. The most important residue is the conserved Lys120, which interacts with the catalytic aspartate (Asp181) of the WPD loop (in its closed conformation) by a hydrogen bond; this interaction stabilizes substrate-bound conformation [38]. Finally, the pTyr loop, which is present in all classical protein tyrosine phosphatases, is considered a pTyr recognition loop and is responsible for the selectivity of pTyr over pSer/pThr [37]. It is characterized by the sequence NXXKNRY, where Tyr46 recognizes the pTyr residue of the substrate and facilitates its access to the active site through electrostatic interactions, Asn44 strengthens the interaction between this residue and active-site residues by forming hydrogen bonds, and Arg45 stabilizes the loop [35,39,40]. All these conserved loops structures are relevant for phosphatase activity and are required for several activities, such as substrate recognition, binding, and catalysis [41].

On the other hand, an additional pocket has been described, called the second aryl phosphate-binding site, B site, or secondary site. This is an important non-conserved site that regulates the substrate specificity [42,43]. It was discovered through an unexpected binding mode observed in the complex with the compound bis-(para-phosphophenyl) methane [43]. The most important residues in this site are Arg24 and Arg254; their guanidinium moieties interact with oxygen atoms of the proximal phosphate and participate in the recognition of diphosphorylated substrates [42].

### 2.2. Allosteric Sites

As previously mentioned, two allosteric sites have been reported in PTP1B: the C-terminal segment (helix α9, residues 367–394) [33] and a site formed by helices α3, α6, and α7 [34]. The first site was identified using a long form of PTP1B (residues 1–393) in a complex with compound MSI-1436 (trodusquemine). NMR studies indicated that the trodusquemine primary binding site is located between residues 367 and 394 in helix α9 [33].

The other site was discovered by crystalizing PTP1B in complex with benzofuran derivatives. This site is a hydrophobic pocket formed by the side chains of Leu192, Phe196, and Phe280 and is located between the α7, α3, and α6 helices. It was observed that when inhibitors interacted with these helices, especially α7, closure of the WPD loop was prevented [34].

## 3. Computational Strategies Applied to Discover PTP1B Inhibitors

A considerable number of PTP1B inhibitors have been reported in recent decades. Many have not only been discovered by experimental procedures but also by using computational methods. Such methods have allowed for the successful development of selective and potent PTP1B inhibitors, in addition to guiding the optimization of different scaffolds [44,45,46,47,48,49]. Molecular docking, virtual screening, pharmacophore modeling, and QSAR are some of the strategies used to this end and will be briefly described hereafter.

### 3.1. Virtual Screening

Virtual screening is a strategy that permits the search for molecules with potential biological activity in large chemical libraries using a computational model [50,51]. Many computational models can be employed to perform virtual screening analysis. They can be categorized as ligand-based and receptor-based virtual screening. Ligand-based methods, for which no information on the receptor is necessary, leverage the information provided by a compound or a set of compounds that is active on the desired target to identify other molecules in the database with similar structural characteristics [52]. On the other hand, receptor-based strategies require knowledge of the 3D structure of the target receptor binding site to select compounds according to their likelihood to bind to the receptor. These involve molecular docking of each ligand to the binding site of the target. In both categories, the obtained information is then used to rank the compounds to select and experimentally evaluate a small subset for biological activity [53]. In addition, the virtual screening approach allows for screening of molecules that do not necessarily exist physically but which can be obtained through purchase or synthesis, enriching ligand libraries and saving time and money [54]. However, this computational approach is incapable of correctly ranking all the molecules in a library or finding all possible active compounds due to the inaccuracy of the scoring functions employed to identify active molecules [55]. The reader is referred to [56,57,58,59] for a comprehensive review.

### 3.2. Molecular Docking

Molecular docking predicts the binding mode of chemical entities within the targeting cavity of the receptor of interest and provides an estimated binding affinity value through a search algorithm and energy-scoring function [60,61,62]. The common steps to carry out this approach are as follows: selection of target and ligand 3D structures, followed by preparation of those structures, depending on the requirements of the molecular docking protocol being employed [63,64]. Three types of docking can be chosen: rigid, semi-flexible, or flexible docking. For rigid docking, both the receptor and ligand are kept in a rigid position [61]. Semi-flexible involves a motionless receptor, whereas ligand flexibility is permitted, which allows for a faster and more direct process [64]. Flexible docking is much more computationally intensive than rigid docking, owing to flexible ligands and receptors [65]. Additionally, the results obtained with this tool can be improved by combining different scoring functions to obtain a consensus score [66]. On the other hand, other scoring functions allow for establishment of a new molecule by screening small compounds (fragments) in a receptor cavity; this is called fragment-based docking. This strategy helps to optimize hits, improving the interaction at the binding site, as well as their physicochemical, pharmacokinetic, and toxicological properties, and new chemical libraries are generated to be synthesized [67]. The reader is referred to [68,69,70] for a comprehensive review.

### 3.3. Pharmacophore Modeling

Another strategy to select molecules from large libraries is pharmacophore modeling, which allows for selection of compounds with similar chemical and physical features, assuming that they have related biological activity [71,72]. A pharmacophore model can be built by superposing a set of active molecules and extracting common chemical features that are necessary for their biological activity; this process is called ligand-based pharmacophore modeling [44]. Moreover, a structure-based pharmacophore modeling strategy can be followed. This consists of obtaining the essential chemical features for the optimal interaction between a biological receptor and a ligand; however, it is necessary to know the receptor 3D structure [44,45]. The reader is referred to [73,74] for a comprehensive review.

### 3.4. QSAR

Quantitative structure–activity relationship (QSAR) is another popular strategy applied to the discovery of active molecules. QSAR consists of building mathematical models that statistically correlate the chemical structure with biological/toxicological properties by regression and classification methods [75,76]. In turn, this method can be categorized into 2D and 3D-QSAR. The 2D strategy calculates and compares compound properties in order to find similar molecules with which the compound is being compared (query molecule) [77]. On the other hand, 3D-QSAR relies not only on chemical structures but also on 3D coordinates of atoms to correlate with biological activity and is divided into alignment-based and alignment-independent techniques [78]. The reader is referred to [79,80,81] for a comprehensive review.

## 4. Development of PTP1B Inhibitors through Computational Approaches

In this section, we describe all the studies performed in the last century using a computational tool as the principal strategy to find PTP1B inhibitors and supported with in vitro studies.

**Figure 3:**
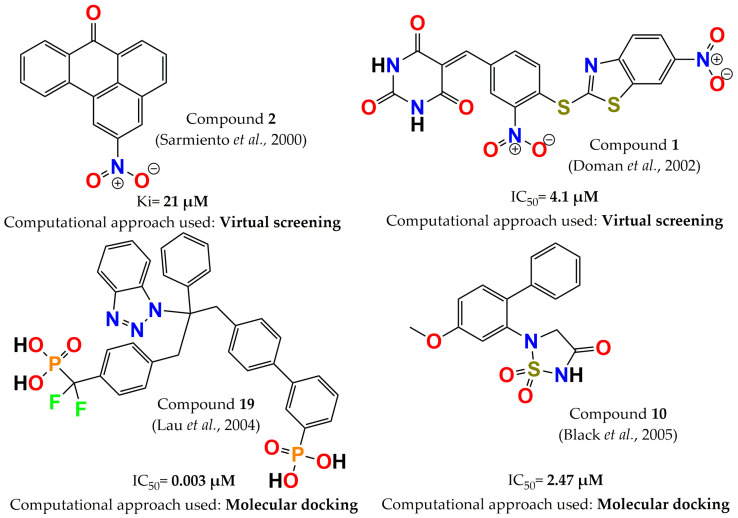
Structure of the most potent PTP1B inhibitors reported between 2000 and 2005 discovered through computational approaches.

In the year 2000, selective PTP1B inhibitors against leukocyte common antigen-related phosphatase (LAR), receptor protein tyrosine phosphatase α (PTPα), and vaccinia H1-related protein phosphatase (VHR) were identified. These inhibitors were discovered by screening approximately 150,000 compounds with a virtual screening approach. From these compounds, twenty-five molecules were selected according to chemical diversity, interactions, overall fit with the enzyme active site, solubility, chemical stability, commercial availability, and cost. Seven compounds showed a high affinity for PTP1B (Ki = 21–510 µM); compound **2** (2-nitrobenzanthrone, Figure 3) was the most potent inhibitor (Ki = 21 µM) and exhibited selectivity against LAR, PTPα, and VHR (threefold higher for PTP1B). Furthermore, this compound was a mixed-type inhibitor [82]. Later, Doman and colleagues compared two methods to identify PTP1B inhibitors: virtual and high-throughput screening (VS and HTS, respectively). In this study, 235,000 and 400,000 compounds were assessed by VS and HTS, respectively. A total of 127 hits from VS and 85 hits from HTS showed IC_50_ values <100 µM. Surprisingly, the most potent molecules were discovered by VS, and the hit rate was enhanced 1700-fold compared to HTS, with compound **1** (Figure 3) being the most notable molecule for its inhibitory activity (IC_50_ = 4.1 µM) [48]. Two years later, another successful case was published by Lau and colleagues. They designed a series of benzotriazole phenyldifluoromethylphosphonic acids through molecular docking on the PTP1B crystal structure. Biphenylphosphonic acid (compound **19**, Figure 3) was the most potent PTP1B inhibitor, with an IC_50_ = 3 nM. In addition, this series of inhibitors was evaluated in several phosphatases, and a moderate selectivity against T-cell protein tyrosine phosphatase (TCPTP) was observed [83]. In 2005, PTP1B inhibitors were designed by optimizing the scaffold 1,2,5-thiadiazolidin-3-one-1,1-dioxide through molecular docking. The analysis indicated that a carbonyl moiety was necessary because it mimics the water-mediated interaction with PTP1B; additionally, the orthogonal orientation between the two rings was significant. These data were corroborated by NMR experiments and enabled identification of compound **10** (Figure 3), the most potent inhibitor (IC_50_ = 2.47 µM) compared with the other designed molecules [84].

**Figure 4:**
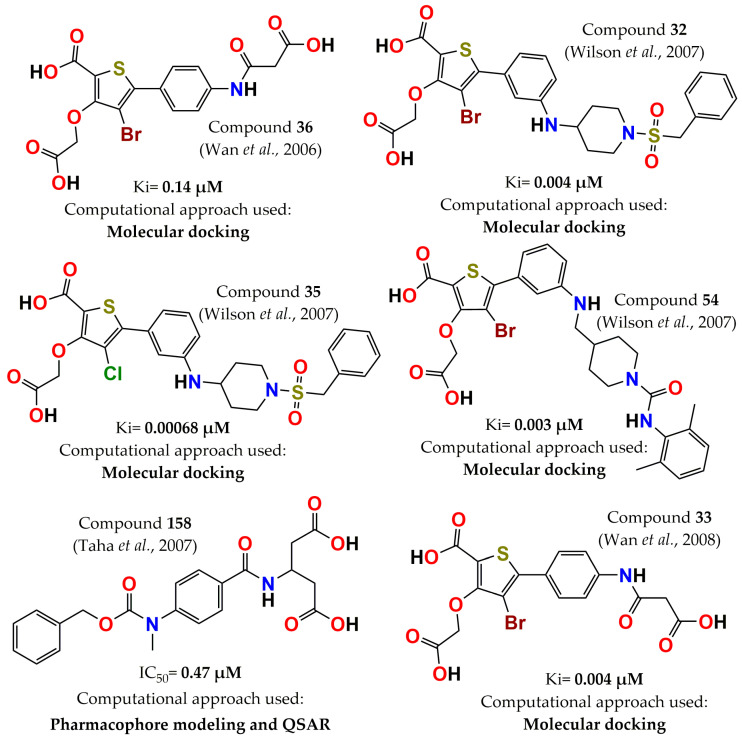
Structure of the most potent PTP1B inhibitors reported between 2006 and 2008 discovered through computational approaches.

The following year, a series of monocyclic thiophenes were designed through the same computational strategy and guided by X-ray cocrystal structural information. It was observed that a hydrogen bond with Asp48 was key to achieving improved inhibition against PTP1B. Therefore, a carboxylic group was incorporated to promote an electrostatic interaction with Arg47, resulting in significant improvement in inhibitory activity (Ki = 0.14 µM, compound **36**, Figure 4) and achieving a 236 and >1000 selectivity ratio against protein tyrosine phosphatase receptor type C (CD45) and LAR, respectively, although selective inhibition vs. TCPTP was not achieved (Ki = 0.18 µM) [85]. Two years later, these authors further pursued the optimization of thiophenes through molecular modeling, considering interactions with Arg24. The structural modification that they included was to remove the N-sulfonyl piperidine, and several side chains were employed to obtain hydrogen bonds with Arg24, which were confirmed by PTP1B-inhibitor crystal structures. The analogous chloro derivative (compound **33**, Figure 4) was the most potent inhibitor (Ki = 4 nM); however, its selectivity was not enhanced relative to TCPTP [86]. Wilson and colleagues optimized compound **3** by molecular modeling, guided by X-ray analysis of the PTP1B-compound **3** complex structure. Compound **35** (5-(3-{[1-(benzylsulfonyl)piperidin-4-yl]amino}phenyl)-3-(carboxymethoxy)-4-chlorothiophene-2-carboxylic acid, Figure 4) was the most active, with subnanomolar activity against PTP1B (Ki = 0.00068 µM), and compound **32** (4-Bromo-3-carboxymethoxy-5-[3-(1-phenylmethanesulfonylpiperidin-4-ylamino)phenyl]thiophene-2-carboxylic acid, Figure 4), with a Ki = 0.004 µM, resulted in significant selectivity against CD45 (Ki = 77 µM) and LAR (Ki = >500 µM) phosphatases. In addition, compound **54** (4-Bromo-3-carboxymethoxy-5-(3-{[1-(2,6-dimethylphenylcarbamoyl)piperidin-4-ylmethyl]amino}phenyl)thiophene-2-carboxylic acid, Figure 4) showed selectivity between PTP1B and TCPTP (three times, Ki = 0.009 µM). Furthermore, pharmacokinetics studies determined that molecule **32** displayed an active glucose uptake mechanism into hepatocytes [87]. In the same year, pharmacophore and QSAR approaches were used to discover potent PTP1B inhibitors. Here, previously PTP1B inhibitors were employed to build a pharmacophoric model. The best binding hypothesis was integrated into a QSAR equation, and it was used to carry out a 3D search query to screen the National Cancer Institute database. Furthermore, the selected hits were filtered according to Lipinski’s rules. The five compounds with the highest ranking were evaluated in vitro, with IC_50_ values from nanomolar to low micromolar (0.47–3.30 µM), with compound **158** (Figure 4) being the most potent [88].

**Figure 5:**
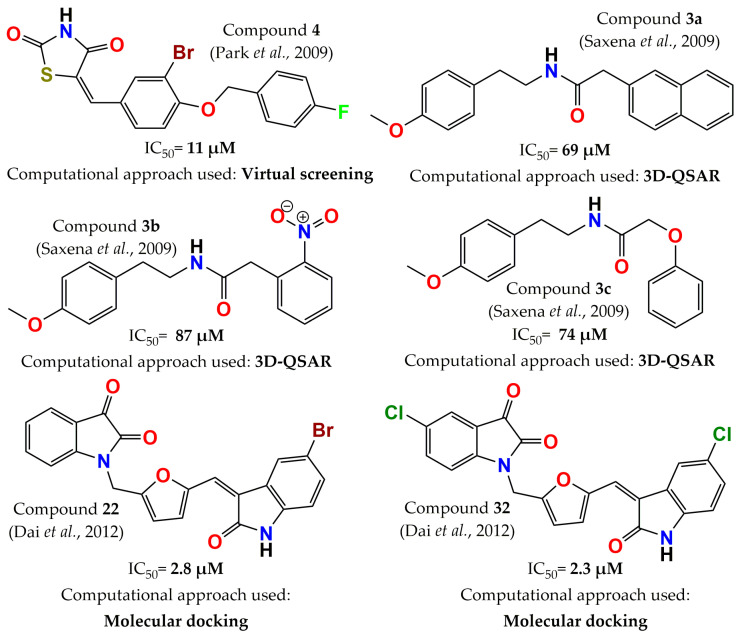
Structure of the most potent PTP1B inhibitors reported between 2009 and 2012 discovered through computational approaches.

In 2009, another study identified new PTP1B inhibitors through virtual screening. The docking library was taken from the latest version of the chemical database distributed by InterBioScreen. The compounds of this library were selected with drug-like filters and without reactive functional groups. This allowed for attainment of a database with 85,000 compounds instead of the 350,000 originals. Additionally, the authors modified the scoring function of the docking software, implementing a new solvation model. The 225 top-scored compounds were evaluated; 21 compounds had more than 90% inhibition at 100 µM, 9 molecules were identified with IC_50_ values ranging from 10 to 50 µM, and compound **4** (thiazolidine-2,4-dione derivative, Figure 5) was found to be the most potent [49]. On the other hand, Saxena and colleagues built a 3D-QSAR model (CoMFA model) in order to obtain three N-[2-(4-methoxy-phenyl)ethyl]ace amide derivatives: compounds **3a** (N-[2-(4-methoxyphenyl)ethyl]-2-naphthalen-1-yl-acetamide, Figure 5), **3b** (N-[2-(4-Methoxyphenyl)ethyl]-2-(2-nitrophenyl)-acetamide, Figure 5), and **3c** (N-[2-(4-Methoxyphenyl) ethyl]-2-phenoxy-acetamide, Figure 5). The model predicted that the order of the inhibitory activity would be **3a** > **3b** > **3c**, and the observed order was found to be similar: **3a** (IC_50_ = 69 µM) > **3c** (IC_50_ = 74 µM) > **3b** (IC_50_ = 87 µM). Furthermore, molecules **3a**, **3b**, and **3c** administered at a dose of 100 mg/kg in two in vivo models decreased blood sugar levels by 25.1%, 19.8%, and 24.6%, in a sucrose-loaded rat model and 21.4%, 17.5%, and 20.6% in a streptozotocin-induced diabetic rat model, respectively [89]. A molecular-docking-guided design allowed for the synthesis of a series of di-indolinone derivatives. This strategy led to the discovery of PTP1B inhibitors with an IC_50_ in the low micromolar range. Compounds **22** (1-[5-(5-bromo-2-Oxo-1,2-dihydroindol-3-ylidenemethyl)furan-2-ylmethyl]-1H-indole-2,3-dione, Figure 5) and **32** (1-[5-(5-chloro-2-Oxo-1,2-dihydroindol-3-ylidenemethyl)furan-2-ylmethyl]-5-chloro-1H-indole-2,3-dione, Figure 5) showed high inhibitory activity (IC_50_ = 2.8 µM and 2.3 µM, respectively). In addition, both molecules displayed selectivity over other homologous PTPs, such as TCPTP, Src homology-2 protein phosphatase-1 (SHP-1), and LAR of least 4 to 43 times, with IC_50_ values for TCPTP of 11.5 and 18.8 µM, respectively, and >100 µM for the other phosphatases [90].

**Figure 6:**
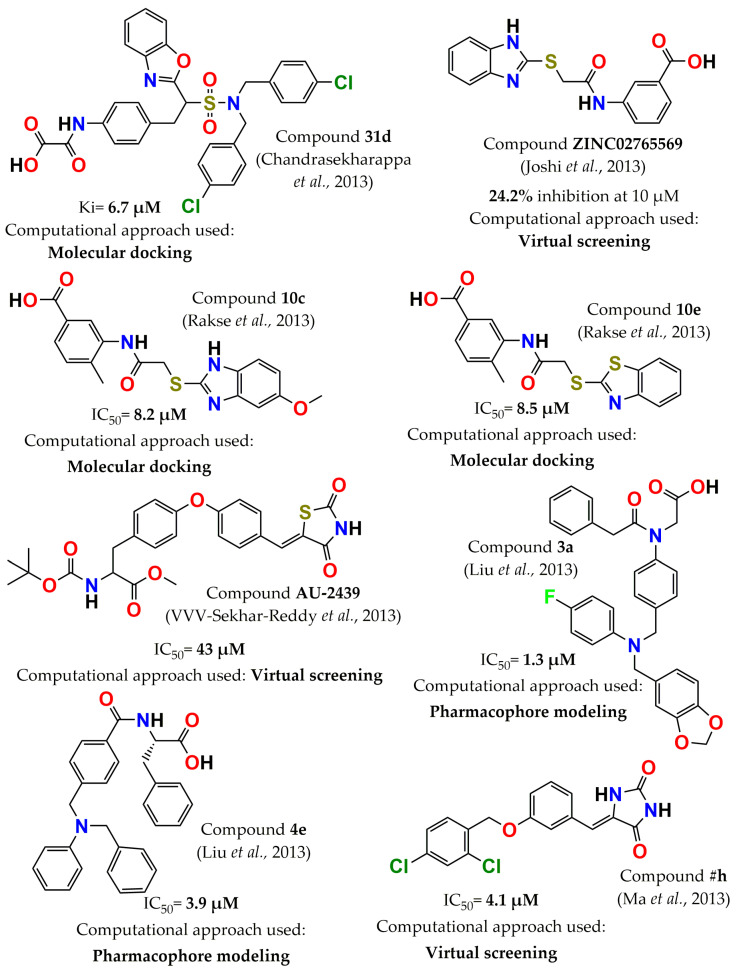
Structure of the most potent PTP1B inhibitors reported in 2013 discovered through computational approaches.

In 2013, various authors reported additional PTP1B inhibitors by employing computational tools. Chandrasekharappa et al. designed a series of benzimidazole and benzoxazole molecules based on molecular docking. The best compounds were selected by employing a flexible docking method, with both PTP1B and TCPTP crystal structures considered. These molecules were synthesized and assessed in PTP1B and TCPTP. Compound **31d** (2-((4-(2-(benzo[d]oxazol-2-yl)-2-(N,N-(4-chlorobenzyl)sulfamoyl)ethyl)phenyl)amino)-2-oxoacetic acid, Figure 6) had the highest affinity for PTP1B (Ki = 6.7 µM), but it was not selective [91]. On the other hand, applying a high-throughput virtual screening strategy using the ZINC and IBS databases, a new inhibitor of PTP1B was discovered: ZINC ID: **ZINC022765569** (3-(2-(1H-benzo[d]imidazol-2-ylthio)acetamido)benzoic acid, Figure 6). This compound inhibited 24% at 10 µM and decreased the glucose uptake by 18% in L6 muscle cells at 50 µM [92]. Furthermore, this inhibitor was taken as a starting point, and through molecular docking studies, a new series of PTP1B inhibitors was developed. Based on the predicted binding mode, different modifications were proposed, such as a methyl substitution at position 5 in the A ring, a substitution in the benzo ring of benzimidazole, and the replacement of the benzimidazole ring by phenyl oxadiazole. Two series of compounds were prepared, and the two most potent compounds, molecules **10c** (3-(2-(5-Methoxy-1H-benzo[d]imidazol-2-ylthio)acetamido)-4-methylbenzoic acid, Figure 6) and **10e** (2-Benzo[d]thiazol-2-ylthio)acetamido)-4-methylbenzoic acid, Figure 6), showed an IC_50_ value of 8.2 and 8.5 µM, respectively [93]. Diphenyl ether derivatives were also identified as PTP1B inhibitors through virtual screening. The study was carried out in both PTP1B and TCPTP using an in-house compound database. From here, forty-three compounds were prioritized based on the docking scores. Compound **AU-2439** (Figure 6) was the most potent, with an IC_50_ of 43 µM. Additionally, it was found to be a selective inhibitor with fivefold selectivity for PTP1B over TCPTP (IC_50_ =230 µM) [94]. In another study, PTP1B inhibitors were discovered via an integrated molecular design strategy of pharmacophore-oriented scaffold hopping based on the template structure of Ertiprotafib. The information obtained from the interaction mode of Ertiprotafib with PTP1B simulated by molecular docking helped to develop a pharmacophore model for Ertiprotafib. Accordingly, twenty-one molecules from five distinct structural classes were designed and synthesized. Of these, nine molecules significantly inhibited PTP1B with a percentage of inhibition higher than 80% at 100 µM. The two most active compounds were **3a** and **4e** (Figure 6), exhibiting an IC_50_ value of 1.3 and 3.9 µM, respectively [46]. Imidazolidine-2,4-dione derivatives were reported as selective PTP1B inhibitors using virtual screening and molecular docking with the core hopping method. The core hopping algorithm helped to find the cores attaching to the scaffold using fragments from the ZINC database. A total of fifty molecules were docked at the active site of PTP1B and TCPTP and; consequently, twelve compounds were synthesized and assessed in both PTP1B and TCPTP. Overall, most molecules were potent and selective, with the compound designed as **#h** (Figure 6) being the most selective, with a selectivity index of almost 34 times PTP1B (IC_50_ =4.1 µM) over TCPTP (IC_50_ > 130 µM) [95].

**Figure 7:**
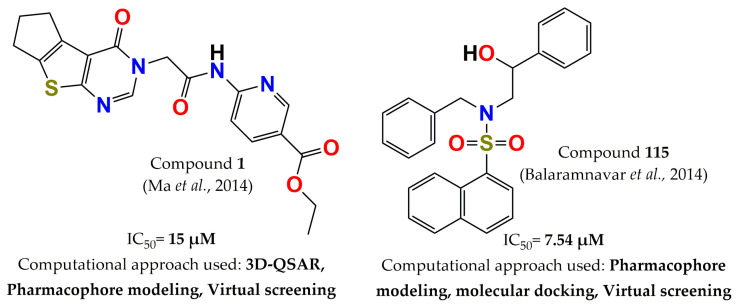
Structure of the most potent PTP1B inhibitors reported in 2014 discovered through computational approaches.

A year later, the same research group designed additional selective inhibitors of PTP1B over TCPTP. In this case, the strategies employed were 3D QSAR, pharmacophore modeling, and virtual screening. All methods were carried out in both enzymes, PTPB and TCPTP, in order to increase the possibility of discovering selective inhibitors. An in-house chemical database was screened, and eight new PTP1B inhibitors were reported. Among these inhibitors, compound **1** (ethyl 6-(2-(4-oxo-4,5,6,7-tetrahydro-3H-cyclopenta [4,5] thieno[2,3-d]pyrimidin-3-yl)acetamido)nicotinate, Figure 7) was the most successful, showing selectivity of eight times for PTP1B (IC_50_ = 15 µM) over TCPTP (IC_50_ > 125 µM) [44]. In the same year, another set of PTP1B inhibitors was identified through pharmacophore modeling, docking, and scaffold-hopping techniques. These approaches allowed for prioritization of ten molecules for synthesis from a library of eighty-six compounds. These compounds inhibited PTP1B in the micromolar range, and characterization of the most potent inhibitor (compound **115**, N-benzyl-N-(2-hydroxy-2-phenylethyl)-2, 4, 6-trimethyl benzene sulfonamide, Figure 7) showed that it improved oral glucose tolerance and enhanced insulin resistance by restoring the insulin level and normalizing the serum lipid profile when assessed in both C57BL/KsJ-db/db mice and an STZ-induced diabetic rat model. Furthermore, this compound augmented the insulin action by modulating the expression of genes involved in insulin signaling, such as IRS 1-2, PI3K, PTPN1, Akt2, AMPK, and PPAR-α. Other experiments corroborated the antiadipogenic effect of this compound on 3T3L-1 cells, as well as its inhibition of lipid accumulation induced by MDI. In addition, it showed a bioavailability of around 10% in rats after 30 mg/kg oral dosing [96].

**Figure 8:**
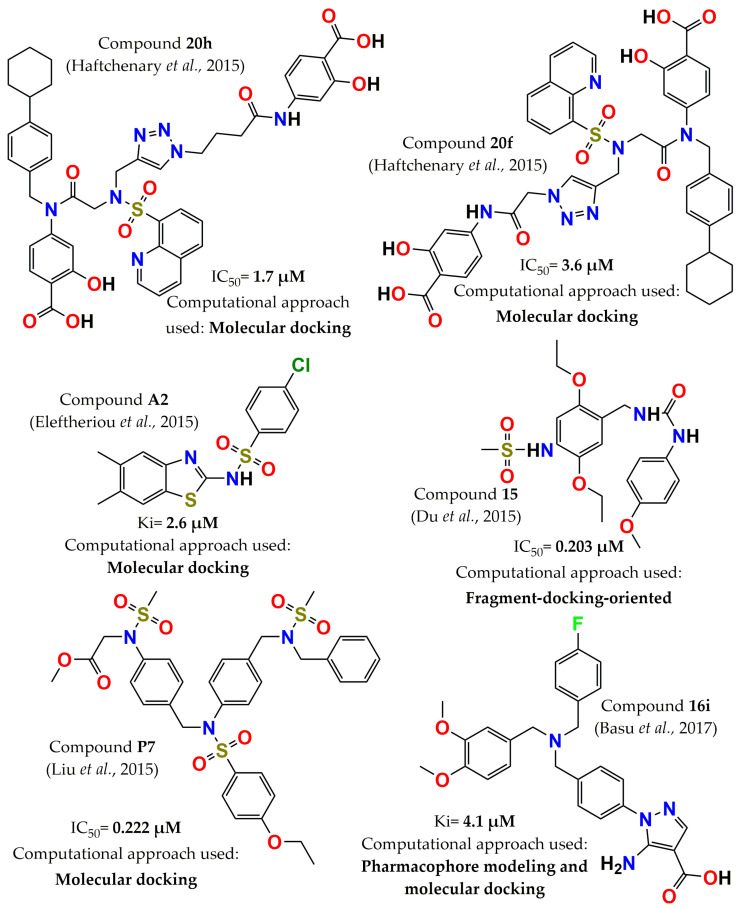
Structure of the most potent PTP1B inhibitors reported between 2015 and 2017 discovered through computational approaches.

In 2015, a salicylic acid derivative was optimized by employing an in silico docking approach. Compound **20h** (4-(4-(4-((N-(2-((4-carboxy-3-hydroxyphenyl)(4-cyclohexylbenzyl)amino)-2oxoethyl)quinoline-8-sulfonamido)methyl)-1H-1,2,3-triazol-1-yl)butanamido)-2hydroxybenzoic acid, Figure 8) exhibited improved potency (IC_50_ = 1.7 µM) compared with the original. Additionally, compounds **20h** and **20f** (4-(2-(4-((N-(2-((4-carboxy-3-hydroxyphenyl)(4-cyclohexylbenzyl)amino)-2oxoethyl)quinoline-8-sulfonamido)methyl)-1H-1,2,3-triazol-1-yl)acetamido)-2hydroxybenzoic acid, Figure 8) showed selectivity for PTP1B over protein tyrosine phosphatase σ (PTPσ) (approximately four to five times). The cytotoxicities of PTP1B inhibitors **20f** and **20h** were determined in Chinese hamster ovary (CHO) cells and showed no toxicity at concentrations of 0.78 to 50 μM. Moreover, Western blot analysis in CHO cells indicated that these three inhibitors increased the levels of autophosphorylation of the insulin receptor (IR). The above results suggest that molecule **20h** represents an interesting lead for further investigation as a PTP1B inhibitor [97]. A fragment-docking-oriented design approach was used to optimize a PTP1B inhibitor that contains a difluoromethylphosphonic acid group. As a result of docking simulations, the phosphoric acid moiety was replaced with a neutral N-(2,5-diethoxy-phenyl)-methanesulfonamide fragment in order to overcome the inconvenience of the negative charge. The IC_50_ values of this molecule and its synthesized analogs were in the nanomolar range. The most potent inhibitor, compound **15**, N-{2,5-Diethoxy-4-[3-(4-methoxy-phenyl)-ureidomethyl]-phenyl}-methanesulfonamide, Figure 8), with an IC_50_ = 203 nM, resulted in a competitive inhibitor. Furthermore, it was observed that it enhanced insulin receptor β phosphorylation and inhibitory activity on TCPTP < 50% at 25 µM [47]. In the same year, thiazolyl derivatives were identified as PTP1B inhibitors through molecular docking analysis in the active and allosteric sites of PTP1B. Four competitive and two allosteric inhibitors with Ki values in the range of 2 to 29 µM were reported, with compound **A2** (Figure 8) being the most active inhibitor [98]. Novel (methanesulfonyl-phenyl-amino)-acetic acid methyl ester analogs were discovered as potent and selective PTP1B inhibitors through detailed analysis of the crystallographic complexes of the inhibitors containing a difluoromethyl phosphonate or carboxymethyl salicylic acid moiety and by applying fragment-based molecular docking. The twelve designed compounds showed inhibitory activity in the nanomolar range; the most potent, compound **P7,** ([(4-{[{4-[(Benzyl-methanesulfonyl-amino)-methyl]-phenyl}-(4-ethoxy-benzenesulfonyl)-amino]-methyl}-phenyl)-methanesulfonyl-amino]-acetic acid methyl ester, Figure 8) had an IC_50_ of 222 nM. Additionally, this molecule was selective against TCPTP (IC_50_ = 1.86 µM) and decreased the dephosphorylation of IRβ in vitro [99]. A new series of amino-carboxylic-based pyrazole derivatives was designed using a structure-based pharmacophore model and molecular docking. In this work, the derivatives were classified into three groups, each with a different hydrophobic tail: 1,2-diphenyl ethanone, oxadiazole, and dibenzyl amines. The oxadiazole derivatives (Ki range of 4-9 µM) and dibenzyl amines (Ki range of 4-11 µM) were the most potent, especially compound **16i** (5-amino-1-[4-[[(3,4-dimethoxyphenyl)methyl-[(4- fluorophenyl), Figure 8). Furthermore, these compounds were stable in rodent liver microsomes, and dibenzyl amine derivatives had better cell permeability in PAMPA than ethanone and oxadiazole derivatives [100].

**Figure 9:**
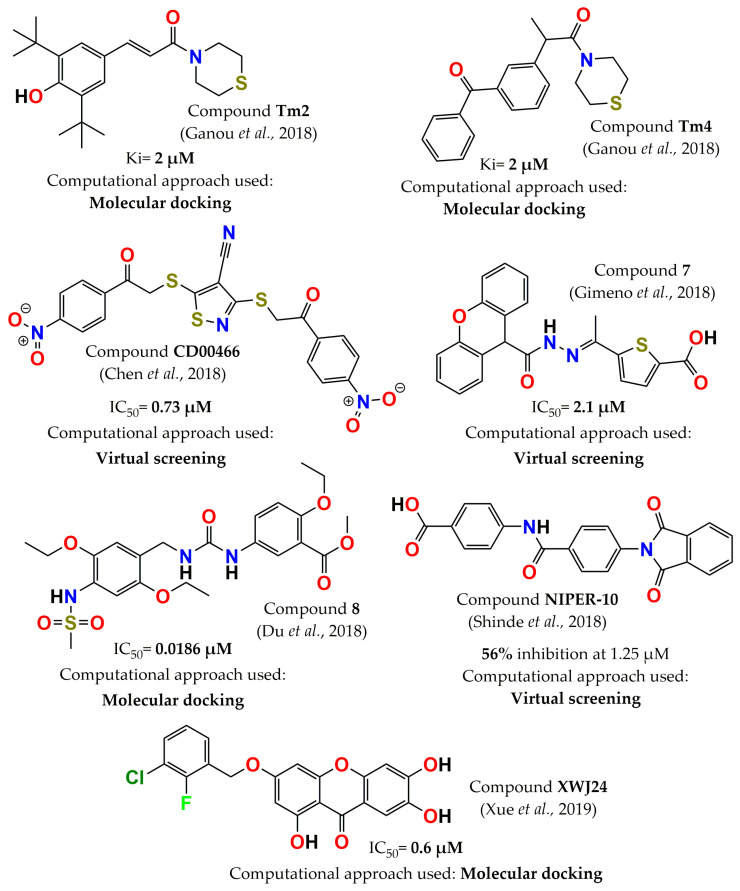
Structure of the most potent PTP1B inhibitors reported between 2018 and 2019 discovered through computational approaches.

In 2018, Ganou et al. continued studying thiazolyl derivatives based on molecular docking analysis of the active and allosteric sites of PTP1B, in addition to considering thiomorpholine derivatives. Most of the compounds were competitive inhibitors (only two were uncompetitive), and the Ki range was between 2 and 23 µM. Compounds **Tm2** and **Tm4** (Figure 9) were the most potent inhibitors of all thiomorpholine and thiazolyl derivatives, with the same Ki value (2 µM). Furthermore, these molecules were selective versus TCPTP (no inhibition on TCPTP at 5 µM) [101]. Three new PTP1B inhibitors were also identified by performing a virtual screening of the Maybridge database. The best inhibitor (compound **CD00466**, Figure 9) exhibited an IC_50_ of 0.73 μM and selectivity of 31-fold over TCPTP (IC_50_ = 22.87 µM) [102]. On the other hand, several filters, such as molecular weight, fingerprints, molecular docking, and electrostatic similarity, were used in a virtual screening protocol. This methodology identified fifteen molecules with IC_50_ values in the range of 1-10 µM; among the molecules with the lowest IC_50_ value was compound **7** (ID: AK-968/41025519 obtained by Specs library, Figure 9). Furthermore, all of these molecules were structurally different, which allowed for exploration of diverse chemical nuclei [103]. In the same year, the structure of compound **15**, designed by Du et al. in 2015 [47], was optimized via a fragment-docking-oriented design, obtaining 11 molecules. The potency was improved, especially in the case of compound **8** (5-[3-(2,5-Diethoxy-4-methanesulfonylamino-benzyl)-ureido]-2-ethoxy-benzoic acid methyl ester, Figure 9), with an IC_50_ value of 18 nM. Furthermore, it increased insulin-stimulated glucose uptake in L6 myotubes and was selective versus four phosphatases; the TCPTP/PTP1B ratio was 35 times (IC_50_ = 670 nM) [104]. Allosteric PTP1B inhibitors were also discovered via virtual screening by employing filters such as Lipinski’s rules of five, potential toxicity, PTP1B and TCPTP pharmacophore-based screening, and molecular docking. Of the 393,932 screened compounds only 23 were selected to be assessed in vitro, and only 10 compounds showed inhibitory activity against PTP1B. The inhibition range was from 10 to 56% at a concentration of 1.25 μM, with compound **NIPER-10** (Figure 9) identified as the most potent [45]. In the following year, potent PTP1B inhibitors were designed via norathyriol optimization. Analysis of the predicted binding mode of norathyriol suggested modifications of four hydroxyl groups at the 1, 3, 6, and 7 positions. The most potent inhibitor, molecule **XWJ24** (3-((3-chloro-2-fluorobenzyl)oxy)-1,6,7-trihydroxy-9H-xanthen-9-one, Figure 9), showed an IC_50_= 0.6 µM. Its characterization revealed that it was a competitive inhibitor and selective against different phosphatases, including TCPTP (IC_50_ = 2.7 µM) [105].

**Figure 10:**
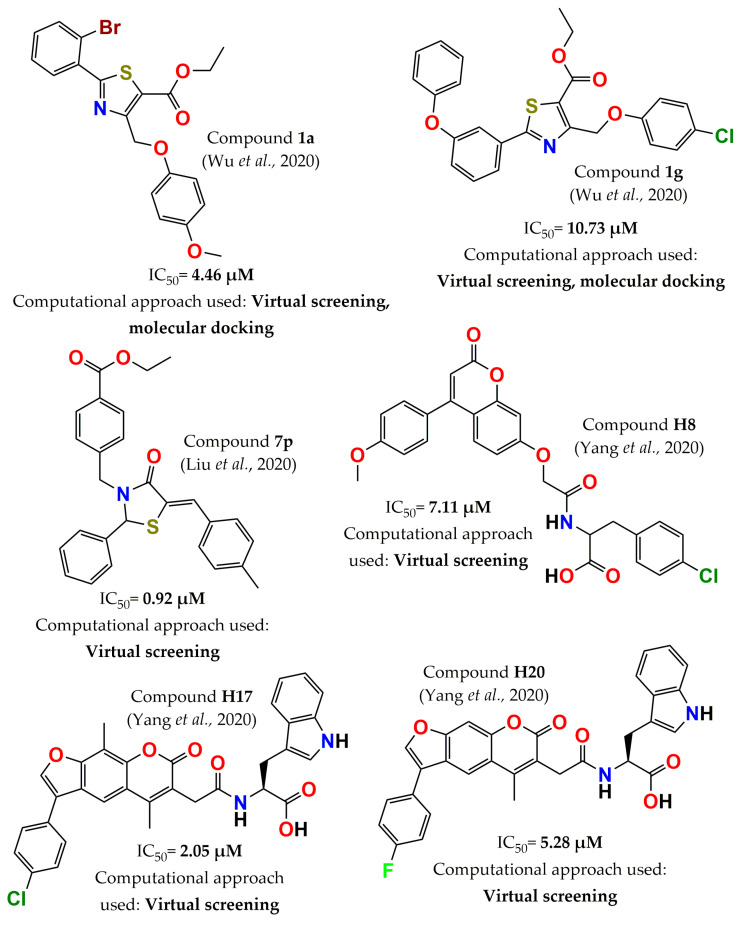
Structure of the most potent PTP1B inhibitors reported in 2020 discovered through computational approaches.

In 2020, Wu and colleagues identified a series of novel PTP1B inhibitors using a virtual screening workflow, molecular docking, and ADMET (absorption, distribution, metabolism, excretion, and toxicity) strategy. Compound **ZINC39276654** was selected from the ZINC database by virtual screening approach to generate one thousand molecule derivatives. Subsequently, one hundred generated derivatives were selected among the top-scored molecules and redocked into the PTP1B active site to choose the top ten compounds (**1a**–**1j**) to evaluate the inhibitory capacity. Compound **1a** (ethyl 2-(2-bromophenyl)-4-((4-methoxyphenoxy)methyl)thiazole-5-carboxylate, Figure 10) exhibited the best inhibitory potency (IC_50_ = 4.46 µM) and acceptable predicted pharmaceutical properties. This molecule was selective for PTP1B over other phosphatases—3 times for TCPTP (IC_50_ = 12.28 µM) and >22 times for the rest—Src homology region 2 domain-containing phosphatase 1 (SHP1), megakaryocyte protein tyrosine phosphatase 2), CDC25B (M-phase-inducer phosphatase 2 (MEG2), and LAR. However, compound **1g** (ethyl 4-((4-chlorophenoxy)methyl)-2-(3-phenoxyphenyl)thiazole-5-carboxylate, Figure 10) showed the strongest selectivity for PTP1B over the TCPTP (>9 times, IC_50_ = >100 µM) [106]. In the same year, a series of seventeen novel 4-thiazolinone derivatives that shared the scaffold of compound **ZINC99459** was discovered by virtual screening. ADMET prediction (including human intestinal absorption, blood–brain barrier, plasma protein binding, aqueous solubility, cytochrome P450 2D6 binding, and toxicity) indicated that these compounds showed good drug-likeness properties. Then, in vitro enzyme inhibitory activity was determined on PTP1B and other phosphatases. Compound **7p** (Figure 10) was identified as the most potent (IC_50_ = 0.92 µM) and selective inhibitor, >100 times (IC_50_ = >100 µM) against TCPTP, SHP2, CDC25B, LAR, and MEG2 and at least 24 times against SHP1 [107]. Another effort to find PTP1B inhibitors via computational tools was a work reported by Yang and colleagues. A natural product library of approximately 122,000 compounds was screened by different in silico filters, such as the 3D-QSAR model, 2D fingerprint similarity, and molecular docking. The twenty-six molecules prioritized by virtual screening were explored to determine their inhibition against PTP1B. The most active compounds, coumarin derivatives, showed an IC_50_ in the micromolar range. Among them, nine molecules were further evaluated against several phosphatases showing selective inhibition for PTP1B over the other phosphatases, including TCPTP. The most active inhibitor was **H17** (IC_50_ = 2.05 µM, Figure 10), but it was not selective (inhibitory activity on TCPTP: 73.5% at 10 µM). The most selective compounds were **H8** and **H20** (Figure 10), with a selectivity of approximately 10 times (inhibitory activity < 10% at 10 µM) with respect to TCPTP, CD45, LAR, and VHR phosphatases [108].

**Figure 11:**
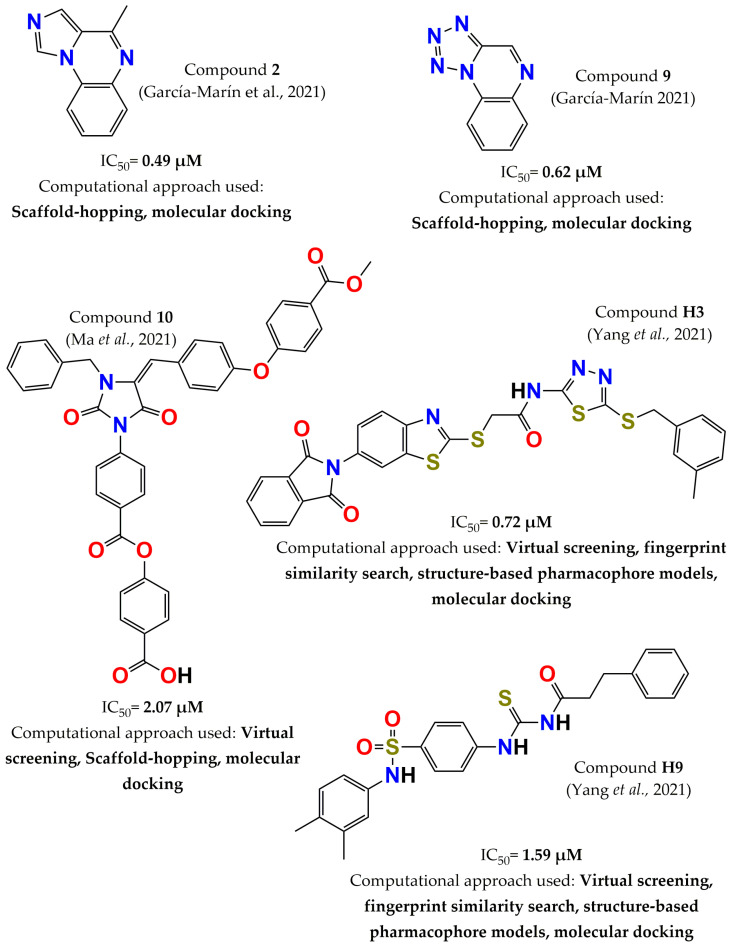
Structure of the most potent PTP1B inhibitors reported in 2021 discovered through computational approaches.

In 2021, the scaffold-hopping approach was employed to optimize the structure of compound **1** based on the replacement of the pyrrole ring by azoles. The new compounds showed better drug-like properties than compound **1** according to the results obtained by means of in silico tools. Most molecules showed good inhibitory activity within the IC_50_ range of 0.46–2.17 µM. Among these compounds, compound **2** (4-methylimidazo[1,2-a]quinoxaline, IC_50_ = 0.49, Figure 11) and compound **9** (tetrazolo[1,5-a]quinoxaline, IC_50_ = 0.62, Figure 11) displayed the highest potency. Additionally, selectivity evaluation of these compounds against TCPTP showed that the most selective was compound **2** (two times, inhibitory activity on TCPTP: 44% at 1 µM). This molecule also increased glucose uptake by 15% relative to control cells in phenotypic models [109]. Ma and colleagues discovered imidazolidine-2,4-dione derivatives as PTP1B inhibitors with structural diversity using virtual screening, scaffold hopping, ADMET prediction, and molecular docking. First, the compound **CHEMBL213560**, obtained by virtual screening, was optimized using the scaffold-hopping method. The designed compounds, with good ADMET results, were used to develop a molecular docking analysis. Fifteen compounds with high scores for PTP1B protein were selected to evaluate their biological activity. These molecules were assessed against PTP1B, Src homology region 2 domain-containing phosphatase 2 (SHP2), and LAR; compound **10** ((E)-4-(methoxycarbonyl)benzyl 4-((3-benzyl-4-(4-((4-(methoxycarbonyl)benzyl)oxy)benzylidene)-2,5-dioxoimidazolidin-1-yl)methyl)benzoate, Figure 11) the highest PTP1B inhibition, with an IC_50_ of 2.07 µM. Moreover, this molecule had 10 times higher inhibitory capacity on PTP1B than SHP2 and 60 times higher than that of LAR [110]. Finally, another study of integrated virtual screening consisting of fingerprint similarity search, structure-based pharmacophore models, and molecular docking was carried out to search for potential allosteric PTP1B inhibitors from commercially available chemical libraries. Of 184,922 molecules, nine compounds were selected to be evaluated in vitro. Two compounds were the most active: **H3** (IC_50_ = 0.72 µM, Figure 11) and **H9** (IC_50_ = 1.59 µM, Figure 11). They were further evaluated against TCPTP and SHP2, displaying selective inhibition of PTP1B over both phosphatases (>60 times, IC_50_ = >100 µM) [111].

## 5. Conclusions

A considerable number of PTP1B inhibitors has been reported as outstanding and potential hits or leads for new drugs against type 2 diabetes. Several potent and selective PTP1B inhibitors have been identified via different computational tools. These studies reported compounds with IC_50_ and Ki values from the nanomolar to the micromolar range. Furthermore, some of these compounds were found to be selective on PTP1B over TCPTP, its closest homolog, with a selectivity range of 2 to 100 times. Moreover, when the characterization was far away, they showed the ability to improve glucose uptake. The PTP1B inhibitor design process remains challenging, which has led to many academic research laboratories and pharmaceuticals organizations continuing the search for effective and safe oral available compounds. Therefore, this review encourages the use of computational techniques and includes helpful information that increases the knowledge generated to date about PTP1B inhibition, with a positive impact on the route toward obtaining a new drug against type 2 diabetes with PTP1B as a molecular target.

## Data Availability

Available data are presented in the manuscript.

## References

[1] WHO (2020). https://www.who.int/.

[2] Garg S., Maurer H., Reed K., Selagamsetty R. (2014). Diabetes and cancer: Two diseases with obesity as a common risk factor. Diabetes Obes. Metab..

[3] Stavrovskaya A. (2000). Cellular mechanisms of multidrug resistance of tumor cells. Biochem. C/C Biokhimiia.

[4] Gewirtz D.A., Bristol M.L., Yalowich J.C. (2010). Toxicity issues in cancer drug development. Curr. Opin. Investig. Drugs.

[5] Inzucchi S.E., Bergenstal R.M., Buse J.B., Diamant M., Ferrannini E., Nauck M., Peters A.L., Tsapas A., Wender R., Matthews D.R. (2012). Management of hyperglycemia in type 2 diabetes: A patient-centered approach: Position statement of the American Diabetes Association (ADA) and the European Association for the Study of Diabetes (EASD). Diabetes Care.

[6] Kalra S., Ghosh S., Aamir A., Ahmed M.T., Amin M.F., Bajaj S., Baruah M.P., Bulugahapitiya U., Das A., Giri M. (2017). Safe and pragmatic use of sodium-glucose co-transporter 2 inhibitors in type 2 diabetes mellitus: South Asian Federation of Endocrine Societies consensus statement. Indian J. Endocrinol. Metab..

[7] Kang J.G., Park C.Y. (2012). Anti-obesity drugs: A review about their effects and safety. Diabetes Metab. J..

[8] Asante-Appiah E., Kennedy B.P. (2003). Protein tyrosine phosphatases: The quest for negative regulators of insulin action. Am. J. Physiol.-Endocrinol. Metab..

[9] Xue B., Kim Y.-B., Lee A., Toschi E., Bonner-Weir S., Kahn C.R., Neel B.G., Kahn B.B. (2007). Protein-tyrosine phosphatase 1B deficiency reduces insulin resistance and the diabetic phenotype in mice with polygenic insulin resistance. J. Biol. Chem..

[10] Cheng A., Uetani N., Simoncic P.D., Chaubey V.P., Lee-Loy A., McGlade C.J., Kennedy B.P., Tremblay M.L. (2002). Attenuation of leptin action and regulation of obesity by protein tyrosine phosphatase 1B. Dev. Cell.

[11] Zabolotny J.M., Bence-Hanulec K.K., Stricker-Krongrad A., Haj F., Wang Y., Minokoshi Y., Kim Y.-B., Elmquist J.K., Tartaglia L.A., Kahn B.B. (2002). PTP1B regulates leptin signal transduction in vivo. Dev. Cell.

[12] Liu H., Wu Y., Zhu S., Liang W., Wang Z., Wang Y., Lv T., Yao Y., Yuan D., Song Y. (2015). PTP1B promotes cell proliferation and metastasis through activating src and ERK1/2 in non-small cell lung cancer. Cancer Lett..

[13] Bjorge J.D., Pang A., Fujita D.J. (2000). Identification of protein-tyrosine phosphatase 1B as the major tyrosine phosphatase activity capable of dephosphorylating and activating c-Src in several human breast cancer cell lines. J. Biol. Chem..

[14] Lessard L., Stuible M., Tremblay M.L. (2010). The two faces of PTP1B in cancer. Biochim. Biophys. Acta-Proteins Proteom..

[15] Stuible M., Doody K.M., Tremblay M.L. (2008). PTP1B and TC-PTP: Regulators of transformation and tumorigenesis. Cancer Metastasis Rev..

[16] Bakke J., Haj F.G. (2015). Protein-tyrosine phosphatase 1B substrates and metabolic regulation. Semin. Cell Dev. Biol..

[17] Zhang S., Zhang Z.Y. (2007). PTP1B as a drug target: Recent developments in PTP1B inhibitor discovery. Drug Discov. Today.

[18] Barr A.J. (2010). Protein tyrosine phosphatases as drug targets: Strategies and challenges of inhibitor development. Future Med. Chem..

[19] Zhang Z.Y., VanEtten R. (1991). Pre-steady-state and steady-state kinetic analysis of the low molecular weight phosphotyrosyl protein phosphatase from bovine heart. J. Biol. Chem..

[20] Andersen J.N., Mortensen O.H., Peters G.H., Drake P.G., Iversen L.F., Olsen O.H., Jansen P.G., Andersen H.S., Tonks N.K., Møller N.P.H. (2001). Structural and evolutionary relationships among protein tyrosine phosphatase domains. Mol. Cell. Biol..

[21] Ibarra-Sánchez M.J., Simoncic P.D., Nestel F.R., Duplay P., Lapp W.S., Tremblay M.L. (2000). The T-cell protein tyrosine phosphatase. Semin. Immunol..

[22] Iversen L.F., Møller K.B., Pedersen A.K., Peters G.H., Petersen A.S., Andersen H.S., Branner S., Mortensen S.B., Møller N.P.H. (2002). Structure determination of T cell protein-tyrosine phosphatase. J. Biol. Chem..

[23] You-Ten K.E., Muise E.S., Itié A., Michaliszyn E., Wagner J., Jothy S., Lapp W.S., Tremblay M.L. (1997). Impaired bone marrow microenvironment and immune function in T cell protein tyrosine phosphatase–deficient mice. J. Exp. Med..

[24] Elchebly M., Payette P., Michaliszyn E., Cromlish W., Collins S., Loy A.L., Normandin D., Cheng A., Himms-Hagen J., Chan C.C. (1999). Increased insulin sensitivity and obesity resistance in mice lacking the protein tyrosine phosphatase-1B gene. Science.

[25] DiMasi J.A., Hansen R.W., Grabowski H.G. (2003). The price of innovation: New estimates of drug development costs. J. Health Econ..

[26] Paul S.M., Mytelka D.S., Dunwiddie C.T., Persinger C.C., Munos B.H., Lindborg S.R., Schacht A.L. (2010). How to improve R&D productivity: The pharmaceutical industry’s grand challenge. Nat. Rev. Drug Discov..

[27] Chernoff J., Schievella A.R., Jost C.A., Erikson R., Neel B.G. (1990). Cloning of a cDNA for a major human protein-tyrosine-phosphatase. Proc. Natl. Acad. Sci. USA.

[28] Frangioni J.V., Beahm P.H., Shifrin V., Jost C.A., Neel B.G. (1992). The nontransmembrane tyrosine phosphatase PTP-1B localizes to the endoplasmic reticulum via its 35 amino acid C-terminal sequence. Cell.

[29] Tonks N.K., Diltz C., Fischer E. (1988). Purification of the major protein-tyrosine-phosphatases of human placenta. J. Biol. Chem..

[30] Lorenzen J.A., Dadabay C.Y., Fischer E.H. (1995). COOH-terminal sequence motifs target the T cell protein tyrosine phosphatase to the ER and nucleus. J. Cell Biol..

[31] Gjörloff-Wingren A., Saxena M., Han S., Wang X., Alonso A., Renedo M., Oh P., Williams S., Schnitzer J., Mustelin T. (2000). Subcellular localization of intracellular protein tyrosine phosphatases in T cells. Eur. J. Immunol..

[32] Barford D., Flint A.J., Tonks N.K. (1994). Crystal structure of human protein tyrosine phosphatase 1B. Science.

[33] Krishnan N., Koveal D., Miller D.H., Xue B., Akshinthala S.D., Kragelj J., Jensen M.R., Gauss C.M., Page R., Blackledge M. (2014). Targeting the disordered C terminus of PTP1B with an allosteric inhibitor. Nat. Chem. Biol..

[34] Wiesmann C., Barr K.J., Kung J., Zhu J., Erlanson D.A., Shen W., Fahr B.J., Zhong M., Taylor L., Randal M. (2004). Allosteric inhibition of protein tyrosine phosphatase 1B. Nat. Struct. Mol. Biol..

[35] Jia Z., Barford D., Flint A.J., Tonks N.K. (1995). Structural basis for phosphotyrosine peptide recognition by protein tyrosine phosphatase 1B. Science.

[36] Barr A.J., Ugochukwu E., Lee W.H., King O.N., Filippakopoulos P., Alfano I., Savitsky P., Burgess-Brown N.A., Müller S., Knapp S. (2009). Large-scale structural analysis of the classical human protein tyrosine phosphatome. Cell.

[37] Tautz L., Critton D.A., Grotegut S. (2013). Protein tyrosine phosphatases: Structure, function, and implication in human disease. Phosphatase Modulators.

[38] Critton D.A., Tautz L., Page R. (2011). Visualizing active-site dynamics in single crystals of HePTP: Opening of the WPD loop involves coordinated movement of the E loop. J. Mol. Biol..

[39] Ren L., Chen X., Luechapanichkul R., Selner N.G., Meyer T.M., Wavreille A.S., Chan R., Iorio C., Zhou X., Neel B.G. (2011). Substrate specificity of protein tyrosine phosphatases 1B, RPTPα, SHP-1, and SHP-2. Biochemistry.

[40] Peti W., Page R. (2015). Strategies to make protein serine/threonine (PP1, calcineurin) and tyrosine phosphatases (PTP1B) druggable: Achieving specificity by targeting substrate and regulatory protein interaction sites. Bioorg. Med. Chem..

[41] Yang J., Niu T., Zhang A., Mishra A.K., Zhao Z.J., Zhou G.W. (2002). Relation between the flexibility of the WPD loop and the activity of the catalytic domain of protein tyrosine phosphatase SHP-1. J. Cell. Biochem..

[42] Puius Y.A., Zhao Y., Sullivan M., Lawrence D.S., Almo S.C., Zhang Z.Y. (1997). Identification of a second aryl phosphate-binding site in protein-tyrosine phosphatase 1B: A paradigm for inhibitor design. Proc. Natl. Acad. Sci. USA.

[43] Low J.L., Chai C.L., Yao S.Q. (2014). Bidentate inhibitors of protein tyrosine phosphatases. Antioxid. Redox Signal..

[44] Ma Y., Jin Y.Y., Wang Y.L., Wang R.L., Lu X.H., Kong D.X., Xu W.R. (2014). The Discovery of a Novel and Selective Inhibitor of PTP 1B Over TCPTP: 3D QSAR Pharmacophore Modeling, Virtual Screening, Synthesis, and Biological Evaluation. Chem. Biol. Drug. Des..

[45] Shinde R.N., Kumar G.S., Eqbal S., Sobhia M.E. (2018). Screening and identification of potential PTP1B allosteric inhibitors using in silico and in vitro approaches. PLoS ONE.

[46] Liu J.Z., Zhang S.E., Nie F., Yang Y., Tang Y.B., Yin W., Tian J.Y., Ye F., Xiao Z. (2013). Discovery of novel PTP1B inhibitors via pharmacophore-oriented scaffold hopping from Ertiprotafib. Bioorg. Med. Chem. Lett..

[47] Du Y., Ling H., Shen J., Li Q. (2015). Discovery of novel, potent, selective and cellular active ADC type PTP1B inhibitors via fragment-docking-oriented de novel design. Bioorg. Med. Chem..

[48] Doman T.N., McGovern S.L., Witherbee B.J., Kasten T.P., Kurumbail R., Stallings W.C., Connolly D.T., Shoichet B.K. (2002). Molecular docking and high-throughput screening for novel inhibitors of protein tyrosine phosphatase-1B. J. Med. Chem..

[49] Park H., Bhattarai B.R., Ham S.W., Cho H. (2009). Structure-based virtual screening approach to identify novel classes of PTP1B inhibitors. Eur. J. Med. Chem..

[50] Shoichet B.K. (2004). Virtual screening of chemical libraries. Nature.

[51] Pradeepkiran J.A., Kumar K.K., Kumar Y.N., Bhaskar M. (2015). Modeling, molecular dynamics, and docking assessment of transcription factor rho: A potential drug target in Brucella melitensis 16M. Drug Des. Devel. Ther..

[52] Sliwoski G., Kothiwale S., Meiler J., Lowe E.W. (2014). Computational methods in drug discovery. Pharmacol. Rev..

[53] Lyne P.D. (2002). Structure-based virtual screening: An overview. Drug Discov. Today.

[54] McInnes C. (2007). Virtual screening strategies in drug discovery. Curr. Opin. Chem. Biol..

[55] Stumpfe D., Bajorath J. (2020). Current trends, overlooked issues, and unmet challenges in virtual screening. J. Chem. Inf. Model..

[56] Cerqueira N.M., Gesto D., Oliveira E.F., Santos-Martins D., Brás N.F., Sousa S.F., Fernandes P.A., Ramos M.J. (2015). Receptor-based virtual screening protocol for drug discovery. Arch. Biochem. Biophys..

[57] Cereto-Massagué A., Ojeda M.J., Valls C., Mulero M., Garcia-Vallvé S., Pujadas G. (2015). Molecular fingerprint similarity search in virtual screening. Methods.

[58] Slater O., Kontoyianni M. (2019). The compromise of virtual screening and its impact on drug discovery. Expert Opin. Drug Discov..

[59] Gimeno A., Ojeda-Montes M.J., Tomás-Hernández S., Cereto-Massagué A., Beltrán-Debón R., Mulero M., Pujadas G., Garcia-Vallvé S. (2019). The light and dark sides of virtual screening: What is there to know?. Int. J. Mol. Sci..

[60] Jain A.N., Nicholls A. (2008). Recommendations for evaluation of computational methods. J. Comput.-Aided Mol. Des..

[61] Guedes I.A., de Magalhães C.S., Dardenne L.E. (2014). Receptor–ligand molecular docking. Biophys. Rev..

[62] Halperin I., Ma B., Wolfson H., Nussinov R. (2002). Principles of docking: An overview of search algorithms and a guide to scoring functions. Proteins.

[63] Pagadala N.S., Syed K., Tuszynski J. (2017). Software for molecular docking: A review. Biophys. Rev..

[64] Morris G.M., Lim-Wilby M., Kukol A. (2008). Molecular docking. Molecular Modeling of Proteins. Methods Molecular Biology™.

[65] Zhou Z., Felts A.K., Friesner R.A., Levy R.M. (2007). Comparative performance of several flexible docking programs and scoring functions: Enrichment studies for a diverse set of pharmaceutically relevant targets. J. Chem. Inf. Model..

[66] Clark R.D., Strizhev A., Leonard J.M., Blake J.F., Matthew J.B. (2002). Consensus scoring for ligand/protein interactions. J. Mol. Graph. Model..

[67] Chen Y., Pohlhaus D.T. (2010). In silico docking and scoring of fragments. Drug Discov. Today Technol..

[68] Torres P.H., Sodero A.C., Jofily P., Silva-Jr F.P. (2019). Key topics in molecular docking for drug design. Int. J. Mol. Sci..

[69] Pinzi L., Rastelli G. (2019). Molecular docking: Shifting paradigms in drug discovery. Int. J. Mol. Sci..

[70] Fan J., Fu A., Zhang L. (2019). Progress in molecular docking. Quant. Biol..

[71] Ke Y.Y., Coumar M.S., Shiao H.Y., Wang W.C., Chen C.W., Song J.S., Chen C.H., Lin W.H., Wu S.H., Hsu J.T. (2014). Ligand efficiency based approach for efficient virtual screening of compound libraries. Eur. J. Med. Chem..

[72] Pradeepkiran J.A., Reddy P.H. (2019). Structure based design and molecular docking studies for phosphorylated tau inhibitors in Alzheimer’s disease. Cells.

[73] Vuorinen A., Schuster D. (2015). Methods for generating and applying pharmacophore models as virtual screening filters and for bioactivity profiling. Methods.

[74] Schaller D., Šribar D., Noonan T., Deng L., Nguyen T.N., Pach S., Machalz D., Bermudez M., Wolber G. (2020). Next generation 3D pharmacophore modeling. Wiley Interdiscip. Rev. Comput. Mol. Sci..

[75] Selassie C., Verma R.P., Abraham D.J. (2003). History of quantitative structure-activity relationships. Burger’s Medicinal Chemistry and Drug Discovery.

[76] Yang G.F., Huang X. (2006). Development of quantitative structure-activity relationships and its application in rational drug design. Curr. Pharm. Des..

[77] Lewis R.A., Wood D. (2014). Modern 2D QSAR for drug discovery. Wiley Interdiscip. Rev. Comput. Mol. Sci..

[78] Cherkasov A., Muratov E.N., Fourches D., Varnek A., Baskin I.I., Cronin M., Dearden J., Gramatica P., Martin Y.C., Todeschini R. (2014). QSAR modeling: Where have you been? Where are you going to?. J. Med. Chem..

[79] Muratov E.N., Bajorath J., Sheridan R.P., Tetko I.V., Filimonov D., Poroikov V., Oprea T.I., Baskin I.I., Varnek A., Roitberg A. (2020). QSAR without borders. Chem. Soc. Rev..

[80] Khan D.A.U. (2016). Descriptors and their selection methods in QSAR analysis: Paradigm for drug design. Drug Discov. Today.

[81] Achary P.G. (2020). Applications of quantitative structure-Activity relationships (QSAR) based virtual screening in drug design: A review. Mini Rev. Med. Chem..

[82] Sarmiento M., Wu L., Keng Y.-F., Song L., Luo Z., Huang Z., Wu G.Z., Yuan A.K., Zhang Z.Y. (2000). Structure-based discovery of small molecule inhibitors targeted to protein tyrosine phosphatase 1B. J. Med. Chem..

[83] Lau C.K., Bayly C.I., Gauthier J.Y., Li C.S., Therien M., Asante-Appiah E., Cromlish W., Boie Y., Forghani F., Desmarais S. (2004). Structure based design of a series of potent and selective non peptidic PTP-1B inhibitors. Bioorg. Med. Chem. Lett..

[84] Black E., Breed J., Breeze A.L., Embrey K., Garcia R., Gero T.W., Godfrey L., Kenny P.W., Morley A.D., Minshull C.A. (2005). Structure-based design of protein tyrosine phosphatase-1B inhibitors. Bioorg. Med. Chem. Lett..

[85] Wan Z.K., Lee J., Xu W., Erbe D.V., Joseph-McCarthy D., Follows B.C., Zhang Y.L. (2006). Monocyclic thiophenes as protein tyrosine phosphatase 1B inhibitors: Capturing interactions with Asp48. Bioorg. Med. Chem. Lett..

[86] Wan Z.K., Lee J., Hotchandani R., Moretto A., Binnun E., Wilson D.P., Kirincich S.J., Follows B.C., Ipek M., Xu W. (2008). Structure-based optimization of protein tyrosine phosphatase-1B inhibitors: Capturing interactions with arginine 24. ChemMedChem.

[87] Wilson D.P., Wan Z.K., Xu W.X., Kirincich S.J., Follows B.C., Joseph-McCarthy D., Foreman K., Moretto A., Wu J., Zhu M. (2007). Structure-based optimization of protein tyrosine phosphatase 1B inhibitors: From the active site to the second phosphotyrosine binding site. J. Med. Chem..

[88] Taha M.O., Bustanji Y., Al-Bakri A.G., Yousef A.M., Zalloum W.A., Al-Masri I.M., Atallah N. (2007). Discovery of new potent human protein tyrosine phosphatase inhibitors via pharmacophore and QSAR analysis followed by in silico screening. J. Mol. Graph. Model..

[89] Saxena A.K., Pandey G., Gupta S., Singh A.B., Srivastava A.K. (2009). Synthesis of protein tyrosine phosphatase 1B inhibitors: Model validation and docking studies. Bioorg. Med. Chem. Lett..

[90] Dai H.-l., Gao L.-X., Yang Y., Li J.-Y., Cheng J.-G., Li J., Wen R., Peng Y.-Q., Zheng J.-B. (2012). Discovery of di-indolinone as a novel scaffold for protein tyrosine phosphatase 1B inhibitors. Bioorg. Med. Chem. Lett..

[91] Chandrasekharappa A.P., Badiger S.E., Dubey P.K., Panigrahi S.K., Manukonda S.R.V. (2013). Design and synthesis of 2-substituted benzoxazoles as novel PTP1B inhibitors. Bioorg. Med. Chem. Lett..

[92] Joshi P., Deora G.S., Rathore V., Tanwar O., Rawat A.K., Srivastava A., Jain D. (2013). Identification of ZINC02765569: A potent inhibitor of PTP1B by vHTS. Med. Chem. Res..

[93] Rakse M., Karthikeyan C., Deora G.S., Moorthy N., Rathore V., Rawat A.K., Srivastava A., Trivedi P. (2013). Design, synthesis and molecular modelling studies of novel 3-acetamido-4-methyl benzoic acid derivatives as inhibitors of protein tyrosine phosphatase 1B. Eur. J. Med. Chem..

[94] VVV-Sekhar-Reddy M., Ghadiyaram C., Kumar-Panigrahi S., Hosahalli S., Narasu-Mangamoori L. (2013). Diphenylether derivative as selective inhibitor of protein tyrosine phosphatase 1B (PTP1B) over T-cell protein tyrosine phosphatase (TCPTP) identified through virtual screening. Mini Rev. Med. Chem..

[95] Ma Y., Sun S.X., Cheng X.C., Wang S.Q., Dong W.L., Wang R.L., Xu W.R. (2013). Design and Synthesis of Imidazolidine-2, 4-Dione Derivatives as Selective Inhibitors by Targeting Protein Tyrosine Phosphatase-1 B Over T-Cell Protein Tyrosine Phosphatase. Chem. Biol. Drug. Des..

[96] Balaramnavar V.M., Srivastava R., Rahuja N., Gupta S., Rawat A.K., Varshney S., Chandasana H., Chhonker Y.S., Doharey P.K., Kumar S. (2014). Identification of novel PTP1B inhibitors by pharmacophore based virtual screening, scaffold hopping and docking. Eur. J. Med. Chem..

[97] Haftchenary S., Jouk A.O., Aubry I., Lewis A.M., Landry M., Ball D.P., Shouksmith A.E., Collins C.V., Tremblay M.L., Gunning P.T. (2015). Identification of bidentate salicylic acid inhibitors of PTP1B. ACS Med. Chem. Lett..

[98] Eleftheriou P., Petrou A., Geronikaki A., Liaras K., Dirnali S., Anna M. (2015). Prediction of enzyme inhibition and mode of inhibitory action based on calculation of distances between hydrogen bond donor/acceptor groups of the molecule and docking analysis: An application on the discovery of novel effective PTP1B inhibitors. SAR QSAR Environ. Res..

[99] Liu P., Du Y., Song L., Shen J., Li Q. (2015). Novel, potent, selective and cellular active ABC type PTP1B inhibitors containing (methanesulfonyl-phenyl-amino)-acetic acid methyl ester phosphotyrosine mimetic. Bioorg. Med. Chem..

[100] Basu S., Prathipati P., Thorat S., Ansari S., Patel M., Jain V., Jinugu R.R., Niranjan S., De S., Reddy S. (2017). Rational design, synthesis, and structure-activity relationships of 5-amino-1H-pyrazole-4-carboxylic acid derivatives as protein tyrosine phosphatase 1B inhibitors. Bioorg. Med. Chem..

[101] Ganou C., Eleftheriou P.T., Theodosis-Nobelos P., Fesatidou M., Geronikaki A., Lialiaris T., Rekka E. (2018). Docking analysis targeted to the whole enzyme: An application to the prediction of inhibition of PTP1B by thiomorpholine and thiazolyl derivatives. SAR QSAR Environ. Res..

[102] Chen X., Gan Q., Feng C., Liu X., Zhang Q. (2018). Virtual screening of novel and selective inhibitors of protein tyrosine phosphatase 1B over T-cell protein tyrosine phosphatase using a bidentate inhibition strategy. J. Chem. Inf. Model..

[103] Gimeno A., Ardid-Ruiz A., Ojeda-Montes M.J., Tomás-Hernández S., Cereto-Massagué A., Beltrán-Debón R., Mulero M., Valls C., Aragonès G., Suárez M. (2018). Combined Ligand-and Receptor-Based Virtual Screening Methodology to Identify Structurally Diverse Protein Tyrosine Phosphatase 1B Inhibitors. ChemMedChem.

[104] Du Y., Zhang Y., Ling H., Li Q., Shen J. (2018). Discovery of novel high potent and cellular active ADC type PTP1B inhibitors with selectivity over TC-PTP via modification interacting with C site. Eur. J. Med. Chem..

[105] Xue W., Tian J., Wang X.S., Xia J., Wu S. (2019). Discovery of potent PTP1B inhibitors via structure-based drug design, synthesis and in vitro bioassay of Norathyriol derivatives. Bioorg. Chem..

[106] Wu J., Ma Y., Zhou H., Zhou L., Du S., Sun Y., Li W., Dong W., Wang R. (2020). Identification of protein tyrosine phosphatase 1B (PTP1B) inhibitors through De Novo Evoluton, synthesis, biological evaluation and molecular dynamics simulation. Biochem. Biophys. Res. Commun..

[107] Liu W.S., Wang R.R., Yue H., Zheng Z.H., Lu X.H., Wang S.Q., Dong W.L., Wang R.L. (2020). Design, synthesis, biological evaluation and molecular dynamics studies of 4-thiazolinone derivatives as protein tyrosine phosphatase 1B (PTP1B) inhibitors. J. Biomol. Struct. Dyn..

[108] Yang Y., Tian J.Y., Ye F., Xiao Z. (2020). Identification of natural products as selective PTP1B inhibitors via virtual screening. Bioorg. Chem..

[109] García-Marín J., Griera M., Alajarín R., Rodríguez-Puyol M., Rodríguez-Puyol D., Vaquero J.J. (2021). A Computer-Driven Scaffold-Hopping Approach Generating New PTP1B Inhibitors from the Pyrrolo [1, 2-a] quinoxaline Core. ChemMedChem.

[110] Ma Y., Ding T.T., Liu Y.Y., Zheng Z.H., Sun S.X., Zhang L.S., Zhang H., Lu X.-H., Cheng X.C., Wang R.L. (2021). Design, synthesis, biological evaluation and molecular dynamics simulation studies of imidazolidine-2, 4-dione derivatives as novel PTP1B inhibitors. Biochem. Biophys. Res. Commun..

[111] Yang Y., Zhang L., Tian J., Ye F., Xiao Z. (2021). Integrated approach to identify selective PTP1B inhibitors targeting the allosteric site. J. Chem. Inf. Model..

